# Analytical data of synthesized deuterated isopropyl myristate and data about the influence of IPM/IPM_deut_ on the thermodynamics and morphology of 2D Stratum Corneum models

**DOI:** 10.1016/j.dib.2017.04.055

**Published:** 2017-05-04

**Authors:** J.S.L. Oliveira, S. Lange, B. Dobner, G. Brezesinski

**Affiliations:** a Max Planck Institute of Colloids and Interfaces, Am Mühlenberg 1, 14476 Potsdam, Germany; b Institute of Pharmacy, Martin Luther University Halle-Wittenberg, Wolfgang-Langenbeck-Strasse 4, 06120 Halle, Germany; c Institute of Medical Physics and Biophysics, University of Leipzig, Härtelstraße 16-18, 04107 Leipzig, Germany

## Abstract

The data in this article shows the effect of isopropyl myristate (IPM) on a 2D *Stratum Corneum* lipid model. In the first part, the analytical characterization of the synthesized deuterated isopropyl myristate is given. Then a BAM image of the pure *Stratum Corneum* model used is shown and a dataset of surface-pressure – area isotherms considering various ratios of deuterated and non-deuterated IPM and the *Stratum Corneum* model mixture is provided. Assuming that after the plateau in the isotherm the area per molecule corresponds only to the *Stratum Corneum* model (squeezing out of IPM), the value of the area will correspond to the percentage of these lipids in the mixture when considering the pure SC model. The comparison of the real and the calculated areas per molecule is also done.

**Specifications Table**

**Table:** 

Subject area	*Physics, Chemistry*
More specific subject area	*Physical-chemistry; Langmuir monolayers*
Type of data	*Tables, figures, images (Brewster Angle Microscopy)*
How data was acquired	*HPLC using a LC Infinity 1200 (Agilent) and for detection a ELSD 2000 (Alltech);*
	*HR-MS using a Proxeon Nano ESI source (Thermo Fisher Scientific) and for detection a LTQ Orbitrap XL Mass Spectrometer (Thermo Fisher Scientific);*
	*ESI-MS using a Finnigan LCQ Classic (Thermo Electron);*
	*NMR using a Agilent Technologies 500 MHz, NB (54 mm) DD2 with the Software VNMRJ4.2;*
	*Surface-Pressure area isotherms using a Langmuir trough (home-made);*
	*Brewster angle microscopy using a BAM2plus (NanoFilm Technologie) equipped with a miniature film balance (NIMA Technology)*
Data format	*Raw in case of BAM; Analyzed for the others*
Experimental factors	*IPM*_*deut*_*dried* in a desiccator under vacuum over P_2_O_5_ before use;
	*Stock solutions were prepared and mixed in the desired ratio for Langmuir monolayer experiments.*
Experimental features	*HPLC chromatogram, HR- and ESI-MS spectra and*^*13*^*C-NMR-spectrum of IPM*_*deut*_*are provided.*
	*Langmuir monolayers of isopropyl myristate, a Stratum Corneum model and mixtures of these two in different ratios are provided, as well as the Brewster angle microscopy image of the SC model.*
Data source location	*HPLC, HRMS and ESI-MS performed at the* Institute of Pharmacy, Martin Luther University Halle-Wittenberg, *Halle, Germany; C-NMR performed at the* Institute of Chemistry, Martin Luther University Halle-Wittenberg, *Halle, Germany; other data are acquired at the Max-Planck Institute of Colloids and Interfaces, Potsdam, Germany*
Data accessibility	*The data is available within this article*

**Value of the data**

• Isopropyl myristate is a common penetration enhancer used in transdermal drug delivery.
• Data on the purification of the new deuterated isopropyl myristate and on the mixture of a 2D Stratum Corneum model with isopropyl myristate are presented.
• Validation of 2D models by direct comparison of deuterated and non-deuterated molecules.

## 1 Data

### 1.1 Analytical characterization of the newly synthesized and purified deuterated isopropyl myristate

**Fig. 1:**
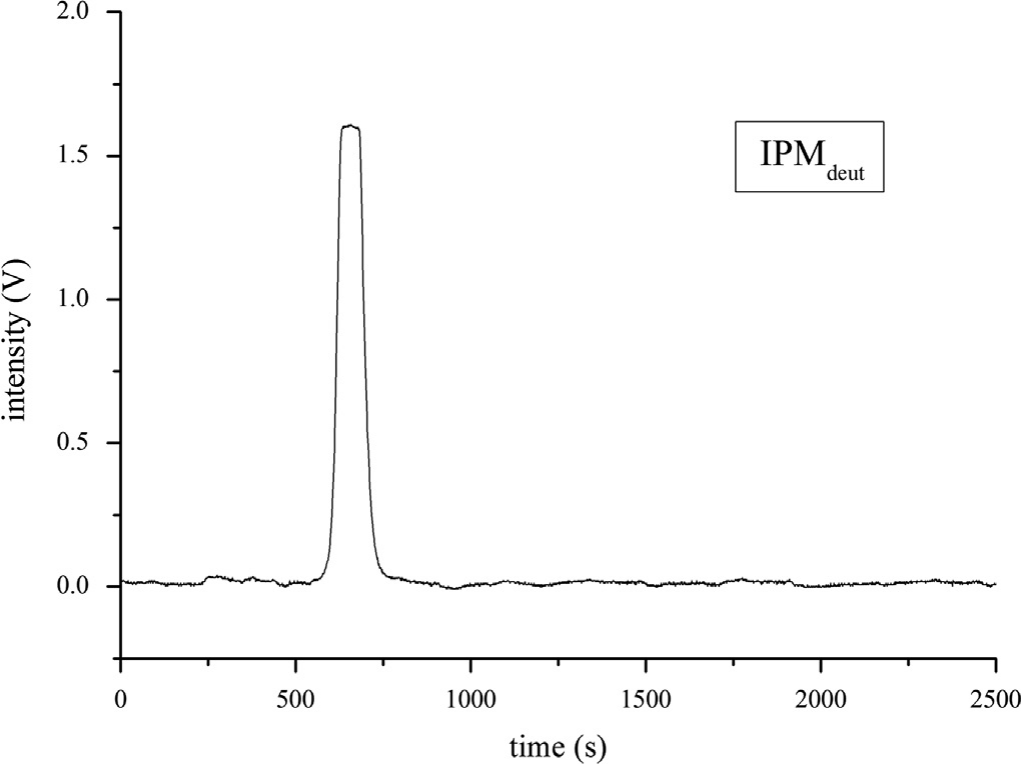
HPLC chromatogram of the purified IPM_deut_.

**Fig. 2:**
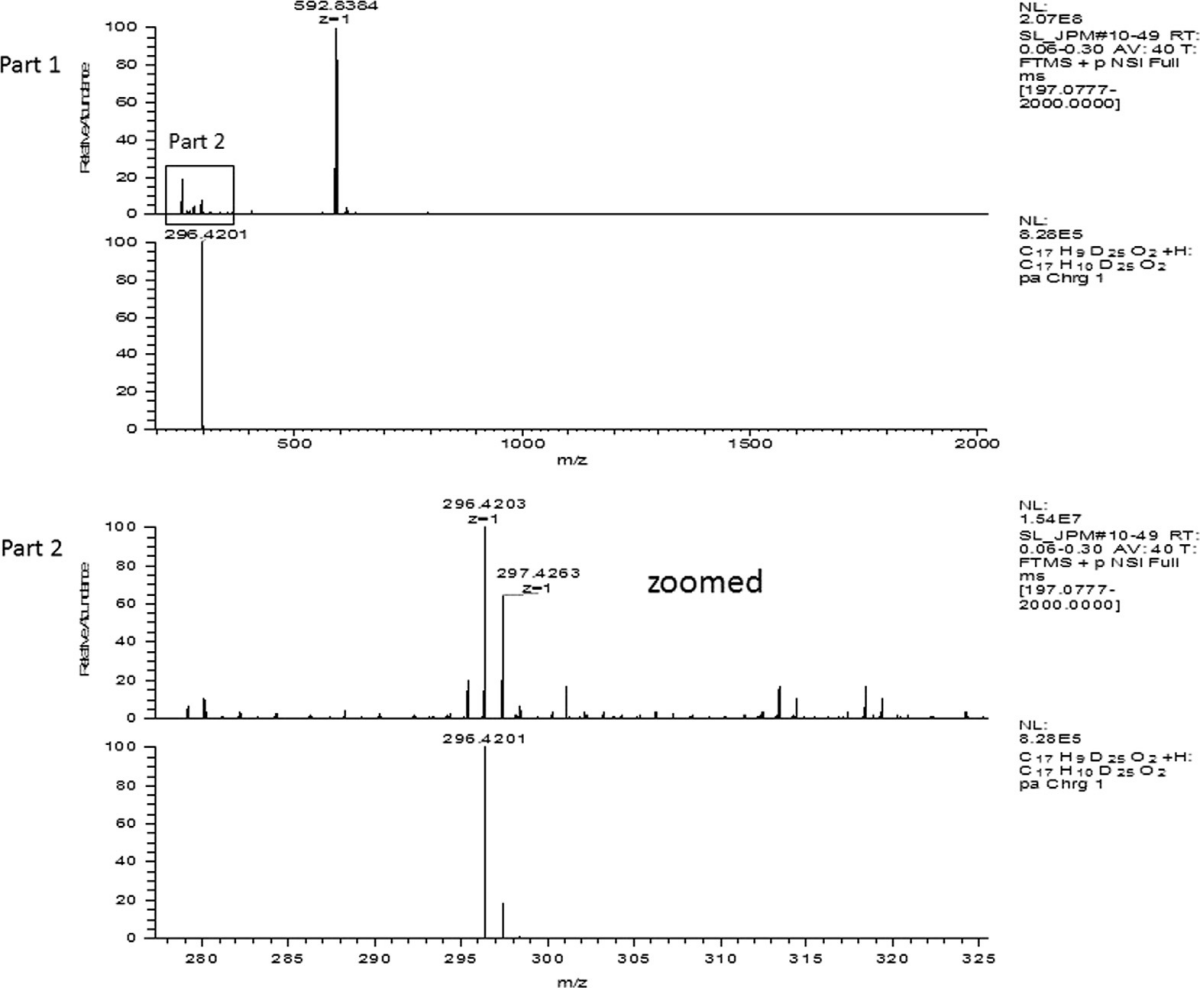
HR-MS spectra of the purified IPM_deut_. Found: 296.4203 [M+H]^+^, 592.8384 [2M+H]^+^; calcd: 296.4201 [M+H]^+^, 592.8402 [2M+H]^+^.

**Fig. 3:**
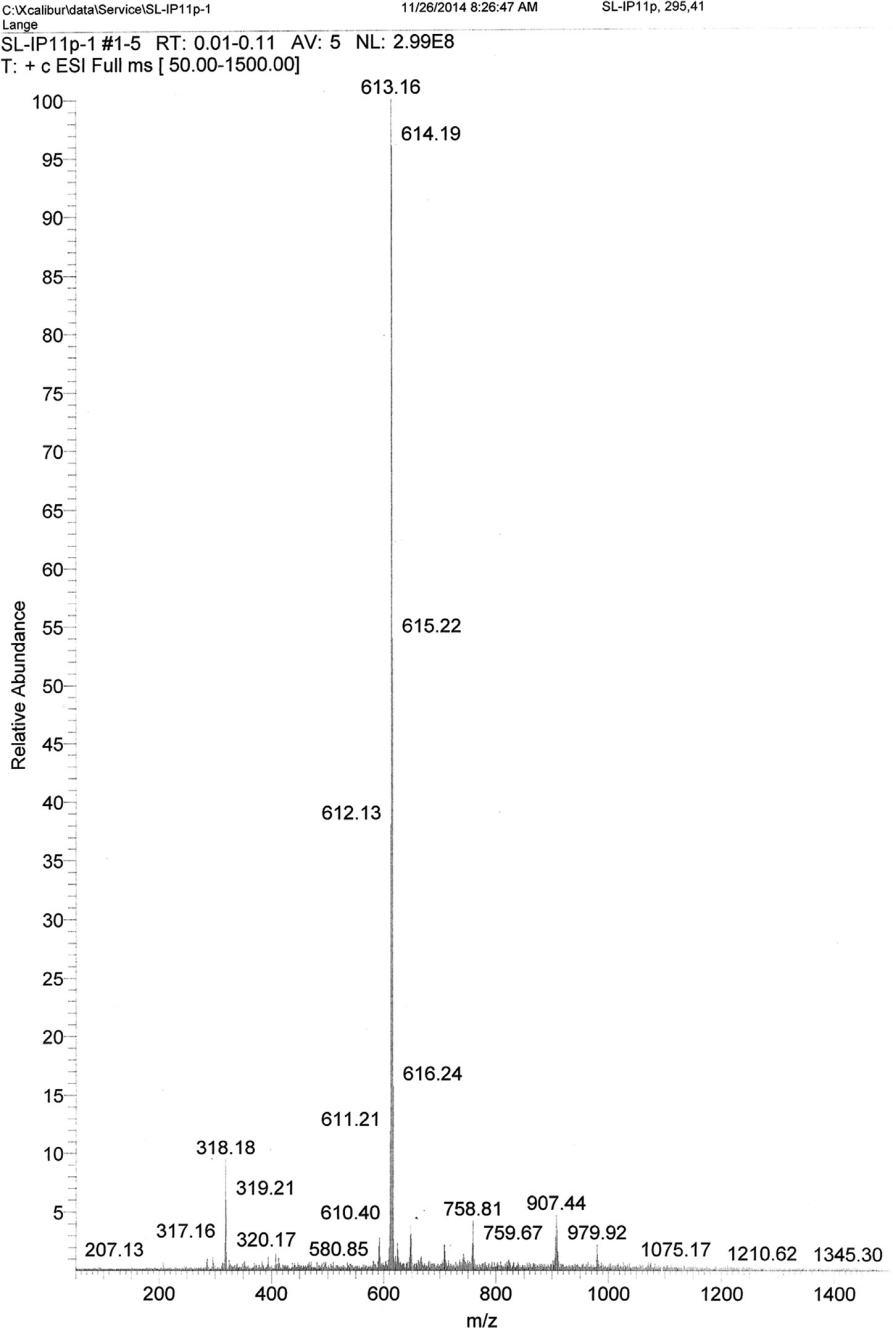
ESI-MS spectra of the purified IPM_deut_. Found: 318.18 [M+Na]^+^, 613.16 [2M+Na]^+^; calcd: 318.41 [M+Na]^+^, 613.83 [2M+Na]^+^.

**Fig. 4:**
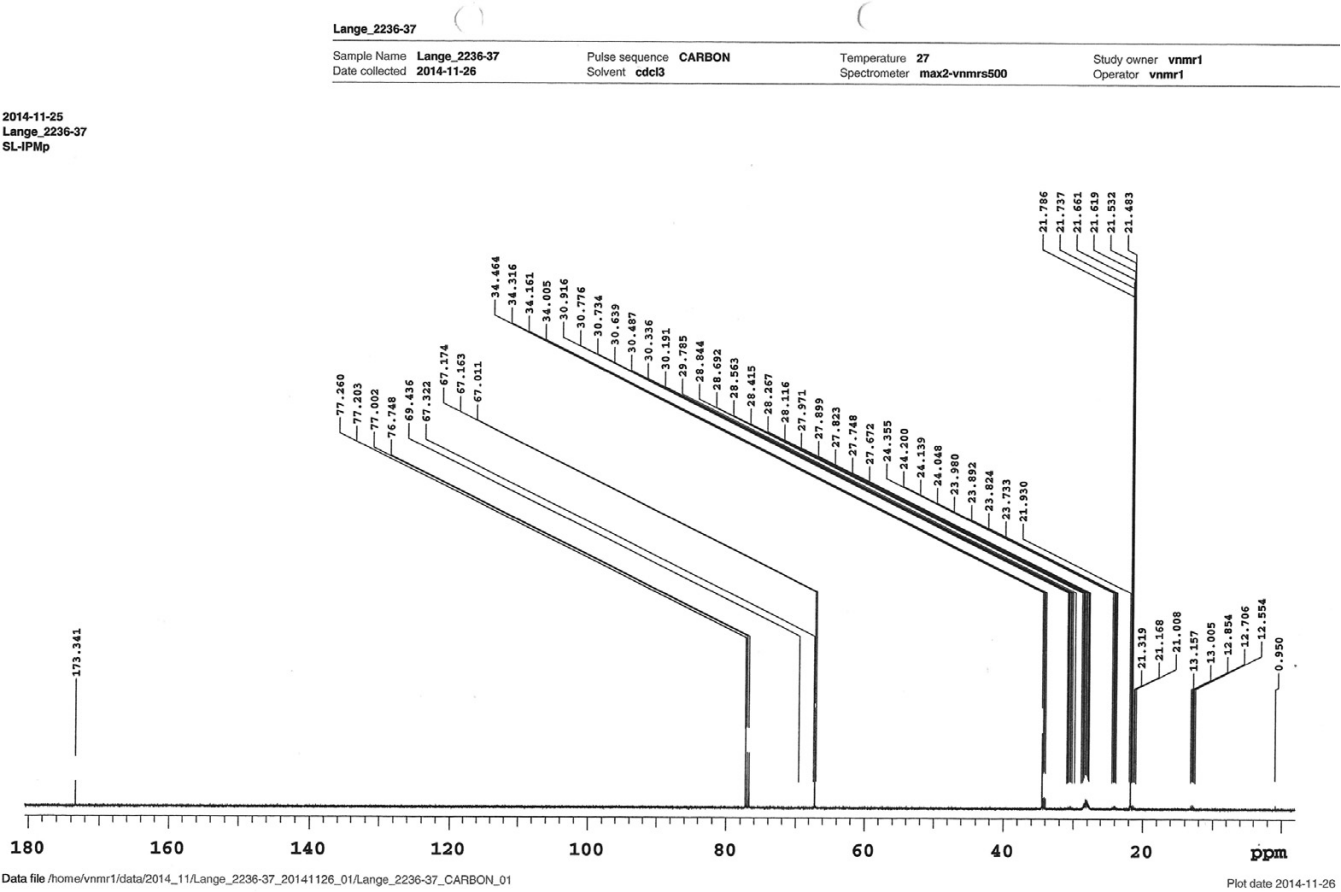
^13^C-NMR-spectrum of the purified IPM_deut_.

The analytical characterization of the newly synthetized and purified deuterated isopropyl myristate was performed by HPLC, HRMS and NMR as shown in Fig. 1, Fig. 2, Fig. 3, Fig. 4.

### 1.2 Data of Langmuir monolayers considering deuterated and non-deuterated isopropyl myristate, *Stratum Corneum* lipid models and their mixtures

**Fig. 5:**
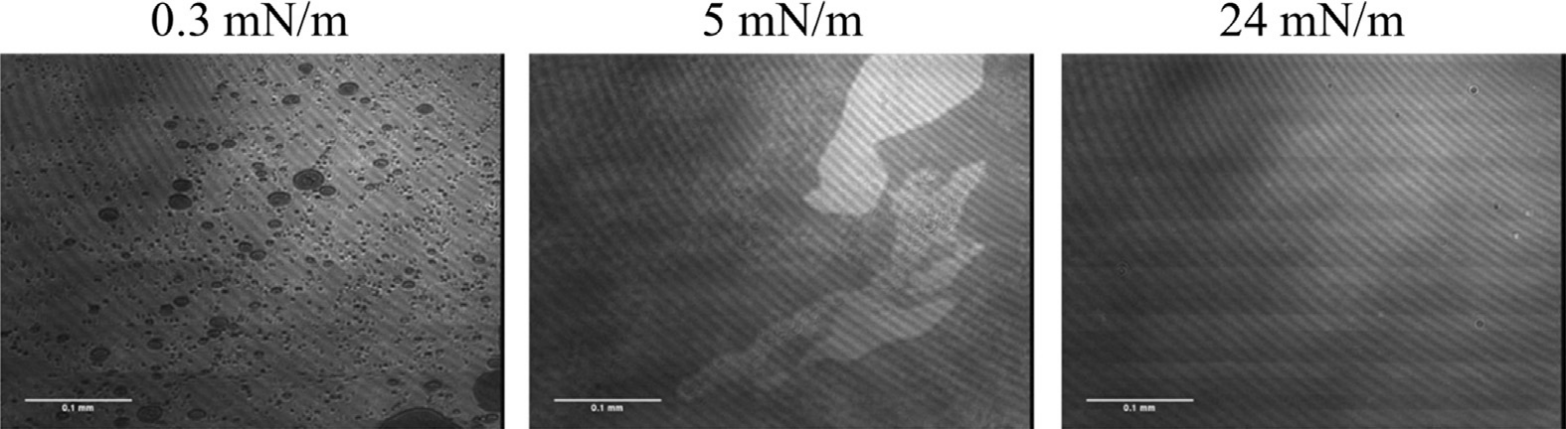
BAM images from the SC model of CER[AP], SA and CHOL in a molar ratio of [1:1:0.7], respectively, on pH 5.5 subphase at 21 °C and selected surface pressures (indicated).

The *Stratum Corneum (SC)* is the outermost layer of our skin and the main problem when addressing topical/transdermal delivery drugs [1]. Langmuir monolayers serve as simple biological models to study the interaction between molecules, as for instance the effect of a penetration enhancer in the SC [2]. As a SC lipid model a mixture of ceramide [AP], stearic acid and cholesterol in a molar ratio of [1:1:0.7] was used. Fig. 5 shows Brewster angle microscopy images from this model at different lateral pressures.

**Fig. 6:**
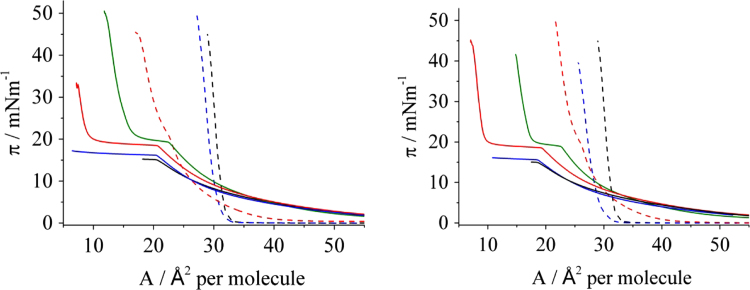
π- A isotherms of IPM (left) and IPM_deut_ (right) and its mixtures with the SC model (ternary mixture of D-form of CER[AP], SA and CHOL in a molar ratio of [1:1:0.7]), at 21 °C, on pH 5.5 subphase. The same colour system in the isotherms is used in both graphs: IPM monolayer (black solid line); SC monolayer (black, dashed line); mixtures of SC model with 10 (blue, dashed line), 30 (red, dashed line), 50 (green solid line), 70 (red solid line) and 90 (blue solid line) mol % IPM.

Ceramide [AP] presents diastereomers: the D-form, which occurs naturally in our skin, and the L-form, which exists only if synthetized. Therefore, a SC model with only the D-form of ceramide [AP] in a mixture with the other two components - which will be called D-SC - is a better model of the human skin lipids. Fig. 6 shows the surface pressure – area isotherms of isopropyl myristate, pure D-SC monolayer and the mixture of the two in different ratios. For comparison and validation reasons the systems with non-deuterated isopropyl myristate (on the left) are compared with the deuterated ones (on the right), and the area per molecule (obtained by averaging the molecular weight of the molecules present in the mixtures) in such isotherms at selected surface pressures is presented in Table 1. In Table 2 the comparison of such areas regarding the calculated area corresponding to the ratio of D-SC present in the mixture is shown.

**Table 1:** Area per molecule taken from the isotherm of the different systems (indicated) considering the D-SC model.

πmN/m	A SC	A SC:IPM_deut_ [90:10]	A SC:IPM [90:10]	A SC:IPM_deut_ [70:30]	A SC:IPM [70:30]	A SC:IPM_deut_ [50:50]	A SC:IPM [50:50]	A SC:IPM_deut_ [30:70]	A SC:IPM [30:70]
	Å^2^	Å^2^	Å^2^	Å^2^	Å^2^	Å^2^	Å^2^	Å^2^	Å^2^
5	37.4	34.5	34.7	34.0	35.1	39.6	39.0	48.3	51.7
10	36.7	33.9	34.0	30.4	31.1	31.3	31.4	34.4	36.7
15	36.2	33.5	33.5	28.5	29.2	27.2	27.6	28.2	29.9
30	35.1	32.6	32.6	25.4	25.9	17.9	18.7	11.9	12.2
39	34.3	31.7		24.2		16.9		11.2	
40	34.2		31.9		24.4		17.5		11.2

**Table 2:** Comparison of the area per molecule taken from the isotherm of the SC and the calculated area corresponding to the percentage of SC present in the mixture (% A SC).

	A SC	A SC:IPM_deut_ [90:10]	A SC:IPM [90:10]	A SC:IPM_deut_ [70:30]	A SC:IPM [70:30]	A SC:IPM_deut_ [50:50]	A SC:IPM [50:50]	A SC:IPM_deut_ [30:70]	A SC:IPM [30:70]
	Å^2^	Å^2^	Å^2^	Å^2^	Å^2^	Å^2^	Å^2^	Å^2^	Å^2^
30 mN/m									

A	35.1	32.6	32.6	25.4	25.9	17.9	18.7	11.9	12.2
% A SC		31.6	31.6	24.6	24.6	17.6	17.6	10.5	10.5
Difference between the areas		1.0	1.0	0.8	1.3	0.3	1.1	1.4	1.7

39 or 40 mN/m									
A	34.3/ 34.2	31.7	31.9	24.2	24.4	16.9	17.5	11.2	11.2
% A SC		30.9	30.8	24.0	23.9	17.2	17.1	10.3	10.3
Difference between the areas		0.8	1.1	0.2	0.5	-0.3	0.4	0.9	0.9

The surface-pressure area isotherms were also measured for the SC model with the racemic mixture of ceramide [AP] (isotherms presented in [3]). Table 3 shows the area per molecule taken from the isotherm of the different systems, while Table 4 shows the comparison of such areas regarding the calculated area corresponding to the ratio of SC present in the mixture.

**Table 3:** Area per molecule taken from the isotherm of the different systems (indicated) considering the SC model with the racemic mixture of CER[AP].

πmN/m	A SC	A SC:IPM_deut_ [90:10]	A SC:IPM [90:10]	A SC:IPM_deut_ [70:30]	A SC:IPM [70:30]	A SC:IPM_deut_ [50:50]	A SC:IPM [50:50]	A SC:IPM_deut_ [30:70]	A SC:IPM [30:70]
	Å^2^	Å^2^	Å^2^	Å^2^	Å^2^	Å^2^	Å^2^	Å^2^	Å^2^
5	31.7	29.2	30.8	32.6	31.0	37.2	39.3	38.9	40.1
10	31.1	28.3	30.0	28.9	26.4	28.7	29.9	27.2	28.6
15	30.7	27.7	29.5	27.7	24.1	24.8	25.3	21.9	23.3
35	30.0	26.5	28.6	23.6	19.8	16.0	14.4	8.5	7.7

**Table 4:** Comparison of the area per molecule taken from the isotherm of the SC and the calculated area corresponding to the percentage of SC present in the mixture (% A SC).

35 mN/m	A SC	A SC:IPM_deut_ [90:10]	A SC:IPM [90:10]	A SC:IPM_deut_ [70:30]	A SC:IPM [70:30]	A SC:IPM_deut_ [50:50]	A SC:IPM [50:50]	A SC:IPM_deut_ [30:70]	A SC:IPM [30:70]
	Å^2^	Å^2^	Å^2^	Å^2^	Å^2^	Å^2^	Å^2^	Å^2^	Å^2^
A	30.0	26.5	28.6	23.6	19.8	16.0	14.4	8.5	7.7
% A SC		27.0	27.0	21.0	21.0	15.0	15.0	9.0	9.0
Difference between the areas		−0.5	1.6	2.6	−1.2	1.0	−0.6	−0.5	−1.3

## 2 Experimental design, materials and methods

Analytical characterization of the newly synthetized and purified deuterated Isopropyl myristate

1. HPLC
  For the HPLC measurement, a stock solution of IPM_deut_ in CH_3_CN with the concentration *of* *1.5* *µg/mL* was prepared. As mobile phase, a mixture of CH_3_CN:MeOH:H_2_O 36;54:10 (v:v:v) was used. The flow rate was 1.2 mL/min with a flow gradient of 100.0 mL/min² and a pressure of 80 bar. The chromatogram was obtained at 265 nm.
2. HR-MS and ESI-MS
  For the measurements, a stock solution of IPM_deut_ in *CHCl*_*3*_*:MeOH 60:40 (V:V)* with the concentration *of* *1.0* *µg/mL* was prepared. The data was collected at 1.3 kV for HR-MS and at 5.0 kV for ESI-MS.
3. ^13^C-NMR
  For the NMR measurement, *10* *µg* *IPM*_*deut*_ *were diluted in* *3* *mL* *CDCl*_*3*_. The spectrum was obtained at 27 °C.

Langmuir monolayers

1. Sample preparation
  Stock solutions of each lipid were prepared in CHCl_3_ (SA, CHOL and IPM or IPM_deut_) or in a CHCl_3_:MeOH mixture [7:3] (w/w) (CER[AP]). Lipid concentrations were in the range of 1 mM. All mixtures were prepared by mixing the respective amount of stock solutions in the desired ratio to a final concentration around 1 mM. To promote mixing, the solution was gently heated, followed by stirring and vortexing for three times. The solutions were spread onto the subphase using a 100 μL Hammilton micro-syringe. 10–15 min were given for the evaporation of the solvent. To mimic the skin surface conditions, a pH 5.5 buffer solution (adjusted with 1 mM HCl) containing 150 mM NaCl and 1 mM EDTA was used as subphase.
2. Brewster Angle Microscopy (BAM)
  A BAM2plus from NanoFilm Technologie (Göttingen, Germany) equipped with a miniature film balance from NIMA Technology (Coventry, UK) was used to image the morphology of the monolayer. The microscope has a frequency-doubled Nd-YAG laser (532 nm, ~50 mW), a polarizer, an analyzer, and a CCD camera. When p-polarized light is directed onto the pure air/water interface at the Brewster angle (~53.1°), zero reflectivity is observed. When a monolayer is added, the light starts to be reflected because of the different refractive index of the surface layer. The reflected light is registered by the CCD camera after passing the analyzer. BAM images of 355×470 µm^2^ were digitally recorded during compression of the monolayer with a resolution of approx. 2 µm.
3. Isotherms
  The pressure-area isotherms were recorded on a computer interfaced home-made Langmuir trough. The trough is equipped with a Wilhelmy balance using a glass plate. The compression speed was 3 Å^2^ molecule^-1^ min^-1^. The temperature was kept constant at (21±0.1) °C. Isotherms were measured three times on individually prepared samples to check for reproducibility.
  The isotherms were plotted in Origin 9 and the area per molecule was read directly from the graph at the surface-pressure considered. The calculated area corresponding to the ratio of SC model present in the mixture was obtained by multiplying the area obtained in the isotherm by the percentage of SC in the mixture. For example, at 35 mN/m the area per molecule of the SC model is 30 Å^2^. Considering the mixture with 90 mol% SC, the calculated area which corresponds to the SC is 27 Å^2^.
