# Supplementary material for: Analytical data of synthesized deuterated isopropyl myristate and data about the influence of IPM/IPMdeut on the thermodynamics and morphology of 2D Stratum Corneum models
Source: Data Brief. 2017 May 4;12:624–31. doi: 10.1016/j.dib.2017.04.055 (PMC5430136; doi:10.1016/j.dib.2017.04.055)
Supplement: Supplementary file 1 — Supporting information [file mmc1.pdf]

# *Conflicts of Interest Statement*

Manuscript title: Analytical data of synthesized deuterated isopropyl myristate and data about the influence of IPM/$IPM_{deut}$ on the thermodynamics and morphology of 2D Stratum Corneum models

The authors whose names are listed immediately below certify that they have NO affiliations with or involvement in any organization or entity with any financial interest (such as honoraria; educational grants; participation in speakers' bureaus; membership, employment, consultancies, stock ownership, or other equity interest; and expert testimony or patent-licensing arrangements), or non-financial interest (such as personal or professional relationships, affiliations, knowledge or beliefs) in the subject matter or materials discussed in this manuscript.

**Author names:**


Oliveira, J.S.L. (Max Planck Institute of Colloids and Interfaces, Am Mühlenberg 1, 14476 Potsdam, Germany)

Lange, S. (Institute of Pharmacy, Martin Luther University Halle-Wittenberg, Wolfgang-Langenbeck-Strasse 4, 06120 Halle, Germany, and Institute of Medical Physics and Biophysics, University of Leipzig, Härtelstraße 16-18, 04107 Leipzig, Germany)

Dobner, B. (Institute of Pharmacy, Martin Luther University Halle-Wittenberg, Wolfgang-Langenbeck-Strasse 4, 06120 Halle, Germany)

Brezesinski, G. (Max Planck Institute of Colloids and Interfaces, Am Mühlenberg 1, 14476 Potsdam, Germany)


The authors whose names are listed immediately below report the following details of affiliation or involvement in an organization or entity with a financial or non-financial interest in the subject matter or materials discussed in this manuscript. Please specify the nature of the conflict on a separate sheet of paper if the space below is inadequate.

**Author names:**

**This statement is signed by all the authors to indicate agreement that the above information is true and correct** (*a photocopy of this form may be used if there are more than 10 authors*):

| Author's name (typed) | Author's signature | Date |
| --- | --- | --- |
| OLIVEIRA, J.S.L. | J.S. Lapa Oliveira | 10 05 17 |
| LANGE, S. | [illegible] | 11 05 17 |
| DOBNER, B | Bodo Dobner | 11 05 17 |
| BREZESINSKI, G | [illegible] | 10 05 17 |
| | | |
| | | |
| | | |
| | | |
| | | |
| | | |
